# Hydrogel Tissue Expanders for Stomatology. Part II. Poly(styrene-maleic anhydride) Hydrogels

**DOI:** 10.3390/polym11071087

**Published:** 2019-06-26

**Authors:** Jakub Hrib, Eva Chylikova Krumbholcova, Miroslava Duskova-Smrckova, Radka Hobzova, Jakub Sirc, Martin Hruby, Jiri Michalek, Jiri Hodan, Petr Lesny, Roman Smucler

**Affiliations:** 1 Institute of Macromolecular Chemistry AS CR, Heyrovsky Sq. 2, 162 06 Prague 6, Czech Republic; 2 Institute of Hematology and Blood Transfusion, U nemocnice 2094/1, 128 20 Prague 2, Czech Republic; 3 1st Faculty of Medicine, Charles University in Prague, Katerinska 32, 121 08 Prague 2, Czech Republic

**Keywords:** hydrogel, self-inflating tissue expander, styrene-maleic anhydride copolymers, swelling, volume expansion, mechanical properties, biocompatibility, in vivo

## Abstract

Self-inflating soft tissue expanders represent a valuable modality in reconstructive surgery. For this purpose, particularly synthetic hydrogels that increase their volume by swelling in aqueous environment are used. The current challenge in the field is to deliver a material with a suitable protracted swelling response, ideally with an induction period (for sutured wound healing) followed by a linear increase in volume lasting several days for required tissue reconstruction. Here, we report on synthesis, swelling, thermal, mechanical and biological properties of novel hydrogel tissue expanders based on poly(styrene-*alt*-maleic anhydride) copolymers covalently crosslinked with *p*-divinylbenzene. The hydrogels exerted hydrolysis-driven swelling response with induction period over the first two days with minimal volume change and gradual volume growth within 30 days in buffered saline solution. Their final swollen volume reached more than 14 times the dry volume with little dependence on the crosslinker content. The mechanical coherence of samples during swelling and in their fully swollen state was excellent, the compression modulus of elasticity being between 750 and 850 kPa. In vitro cell culture experiments and in vivo evaluation in mice models showed excellent biocompatibility and suitable swelling responses meeting thus the application requirements as soft tissue expanders.

## 1. Introduction

The soft tissue expanders (TE) prepared from swelling gels represents a useful modality in reconstructive surgery especially in the maxillofacial area. The surgical use of bone grafts (e.g., alveolar bone graft) is essential in the treatment of many pathologies, but it is often difficult due to periosteal deficiency that often leads to the necessity of grafting soft tissue prior to bone surgery. Keeping the living tissue under a constant tension created by TE leads to the growth of cells and tissue [1]. The periosteum is responsible for the bone growth and its presence on the surface is crucial for bone reconstruction [2]. The use of subperiosteal TE is able to produce excess periosteal tissue and lead to success in clinical setting [3]. TE have many advantages: They allow better wound closure without compromising the blood supply of the flap, they help to augment the bone and their transfer is safer due to higher vascularization [4,5].

The history of TEs is dating back to 1957, to the first use of a rubber silicone balloon to expand skin for ear reconstruction [6]. Until today, TEs have undergone considerable development from balloon-type silicone TEs filled with air or saline solution requiring sequential inflating by injection to self-inflating TEs filled by hypertonic solution enabling swelling due to osmotically driven water uptake [7]. The new area in tissue expansion began with the introduction of osmotically driven volume expansive synthetic hydrogel TE based on hydrophilic polymers such as poly(vinylpyrrolidone) or derivatives of poly(methacrylic acid) [8] which have already been tested in clinical practice [3,9,10,11,12,13,14]. In 2011 the application of self-inflating osmotic hydrogel TE in 24 sites was shown to be successful for vertical ridge augmentation with regard to clinical and histological outcomes and complications [15]. Despite the fact that self-inflating hydrogel TE represents a promising technology, some limitations of its clinical applications exist. Typically, the hydrogel expansion rate is too fast at the initial stage of swelling process, reaching mostly 60%–80% of maximal expansion volume within few hours followed by a smaller gradual volume increase. However, change in the chemical composition of hydrogels mostly does not lead to a reduction of the initial swelling rate, but to an influence on final volume expansion. This was overcome by enclosing the hydrogel into semipermeable silicone membrane with various porosity to control the fluid rate and consequent TE expansion while providing the same swelling capacity [16]. However, the necessity of using an outer membrane constitutes a risk of membrane rupture or obstacles in sizing and shaping the samples as required by the application. So, the preparation of hydrogel TE without any perforated membrane with a suitable swelling pattern meeting the medicinal requirements of biocompatibility and capability to be easily handled still remains as a challenge.

In our previous study [17] we presented the synthesis of TEs in form of a solid and uniform hydrogel blocks without any membrane cover based on poly(2-hydroxyethyl methacrylate-*co*-methacrylic acid) copolymers. These hydrogels exhibited a close-to-linear volume expansion rates up to 3 to 4.5 times of their original volume using a combination of non-degradable and hydrolytically degradable crosslinkers, allowing the volume expansion even in a later stage due to the degradation of the polymer network accompanied by the additional increase in swelling. However, the initial expansion rate was still relatively fast concerning the surgical application. So, the aim of our present work is to prepare the hydrogel TE possessing the delayed and protracted initial swollen volume growth providing sufficient time to allow wound healing after TE implantation and to avoid the risk of rupturing the sutured site.

Copolymers of styrene and maleic anhydride have a long history dating back to 1940s from surfactants, compatibilizers in plastic engineering, epoxy hardeners, separation membranes [18,19]. More recently, these copolymers have been examined in various biomedical applications [20,21] as they exhibit favorable biological behavior and general biocompatibility [22]; e.g., they have been used as models of lipid bilayers or as components for polymer-drug conjugate delivery systems reaching clinical practice under the name SMANCS [23]. However, their application as a tissue expander has not yet been reported.

In the present paper, we designed and synthetized hydrogels based on poly(styrene-*alt*-maleic anhydride) copolymers (polySMA) via radical copolymerization using *p*-divinylbenzene as a styrene-type crosslinker to obtain macromolecular networks. The course of free swelling of polySMA copolymers in aqueous physiological solutions was studied as well as their swelling in vivo under spatial constraints. The mechanical behavior of equilibrium swollen polySMA hydrogels was investigated under compression mode. The thermal properties were monitored by DSC for the dry polySMA copolymers. Finally, in vitro biocompatibility with cell cultures and in vivo performance of intramuscularly implanted hydrogels in mice were investigated, showing excellent biocompatibility and the expected protracted swelling profiles thus providing to be promising candidates for the development of the novel type of soft hydrogel TE.

## 2. Materials and Methods

### 2.1. Materials

Styrene, maleic anhydride (MA), *p*-divinylbenzene (DVB) and 2,2’-azo-bis(isobutyronitrile) (AIBN) were purchased from Sigma-Aldrich (Prague, Czech Republic). Styrene was distilled, MA was resublimed and AIBN was recrystallized from methanol prior to use. Ethyl acetate, ethanol and acetone of analytical grade were obtained from Lach-Ner (Neratovice, Czech Republic). Acetonitrile (ACN) and water of HPLC grade were purchased from VWR (Prague, Czech Republic). Phosphate buffered saline (PBS) solution (pH 7.4) was prepared from tablets supplied by Sigma-Aldrich (Prague, Czech Republic) according to the manufacturer’s instructions. Fetal bovine serum (FBS) was purchased from Sigma-Aldrich (Prague, Czech Republic). Cell culture medium (CellGRO) and AlamarBlue Assay were purchased from ThermoFisher Scientific (Prague, Czech Republic), 24-well plates were from TPP (Prague, Czech Republic).

### 2.2. Preparation of Hydrogels

The copolymers of styrene and MA were prepared by free-radical crosslinking co-polymerization. DVB crosslinker was used in various contents. Ethyl acetate was selected as a thermodynamically good and inert solvent that dissolves styrene and MA monomers and provides homogeneous polymerization mixture as well as the reaction product. The polymerization was initiated thermally using AIBN as an initiator.

**Figure 1:**
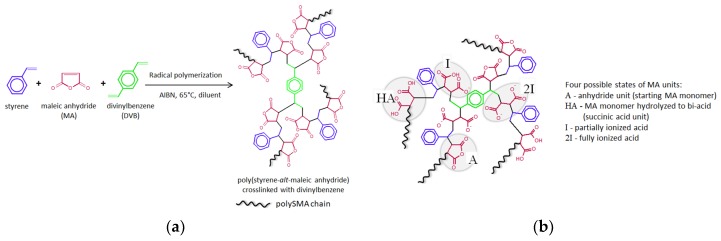
(**a**) Simplified reaction scheme of the synthesis and structure of polySMA hydrogels; (**b**) chemical structure of hydrolyzed polySMA hydrogel after swelling in PBS solution (pH 7.4).

The polymerization mixture consisting of monomers styrene and MA (molar ratio 1:1), crosslinker DVB (0.5, 0.7, and 1.0 mol %), initiator AIBN (0.5 wt %) and ethyl acetate (65 wt %) was homogenized, bubbled by nitrogen, and put into the polyethylene tubes of length 20 cm with an internal diameter of 3 mm. The filled tubes were sealed to avoid the access of oxygen. The polymerization was performed in a thermostatic bath at 65 °C for 16 h. After polymerization, the materials were removed from the tubes and cut into samples of approx. 8 mm in length. The samples were washed in excess of acetone to remove ethyl acetate and possible unreacted residues for five days while exchanging acetone twice a day. Washed samples were dried at 70 °C in a vacuum oven overnight. The scheme of hydrogel preparation is shown in Figure 1a, wherein the samples are coded as polySMA-X, where X designates the content of the crosslinker (0.5, 0.7 or 1.0).

For the determination of thermal and mechanical properties, the hydrogels were prepared in the form of sheets of 6 mm in thickness. The polymerization mixture was injected into a mold formed by a glass plate, a silicone rubber distance frame (6 mm), and a polypropylene plate, all firmly closed with screw clamps, and then the polymerization was performed in a thermostatic bath in temperature mode 1 h at 40 °C, 1 h at 45 °C, and 14 h at 50 °C. The obtained samples were washed as described above.

### 2.3. Characterization of Hydrogels

#### 2.3.1. Swelling Measurement

Polymerized, washed and dried samples (approx. 8 mm in length that was 0.1 g in weight) were weighed and their dimensions were exactly measured using a digital caliper with a resolution of 0.01 mm. Then, the samples were put into 20 mL of PBS solution at room temperature. Sizes and weights were recorded regularly each day for 40 days. The swelling courses are expressed as volume swelling ratio *V*_t_/*V*_0_, where *V*_t_ is the volume at particular time and *V*_0_ is the initial volume of dry extracted sample. All experiments were made in triplicates and average values and standard deviations were calculated (*n* = 3). Swelling in FBS was tested using identical procedure as in PBS.

The parameter *φ*_2_ expresses the equilibrium volume fraction of the polymer in a swollen gel, i.e., it describes the swollen system in a state when the sample is not changing its volume any more (at a given temperature and solute composition) and is calculated according to Equation (1)

(1) $$\varphi_{2}=\frac{V_{nt}}{V_{nt}+V_{s}}$$

where *V*_nt_ is the volume of polymer in equilibrium swollen sample, and *V*_s_ is the volume of the solvent in that swollen gel.

#### 2.3.2. Determination of Crosslink Density

The term “crosslink density” of a macromolecular network denotes the concentration of the elastically active network chains (EANC) per volume element and is typically expressed in moles of EANC per cm^3^ of the macroscopic crosslinked sample [24]. In order to compare crosslink densities of various systems, the EANC concentration should be related to the defined reference state. In our case, the reference state was the dry hydrogel. The nominal crosslink densities of hydrogels *ν*_id_ were calculated from the initial composition of the polymerization mixture according to Equation (2),

(2) $$\nu_{id}=\frac{f\times C}{2}$$

where *f* is the functionality of the crosslinker (for DVB it is four) and *C* is a concentration of network nodes—i.e., moles of crosslinker molecules in a volume element of the monomeric mixture: Thus, related to the network dry state. The Equation (2) is neglecting the volume change caused by polymerization.

The experimentally obtained values of crosslink densities *ν*_e_ were calculated from moduli obtained by compression measurements according to Equation (3),

(3) $$\nu_{e}=\frac{\sigma }{RT{\left(\frac{\varphi_{0}}{\varphi_{2}}\right)}^{\frac{2}{3}}\varphi_{2}\left(\lambda -\lambda^{-2}\right)}$$

where *σ* is the applied stress [Pa·m^−2^], *R* is the universal gas constant [8.14 J·mol^−1^·K^−1^], *T* is the temperature during measurements [K], *φ*_2_ is the volume fraction of the polymer network in the swollen sample, *φ*_0_ is the polymer volume fraction of the gel components during polymerization and *λ* is the relative deformation of the specimen (L_0_/L).

#### 2.3.3. Mechanical Properties

The mechanical properties of equilibrium swollen polySMA hydrogels were characterized by uniaxial compression measurements. The testing was done using a universal tensile apparatus Instron model 6025/5800 R (Instron Limited, UK) equipped with a liquid chamber, a parallel plate geometry and a load cell 100 N. During the compression loading, the equilibrium swollen disk-shaped samples of height approx. 6 mm and diameter approx. 17 mm were immersed in a temperature conditioned liquid chamber and loaded at a room temperature (23 ± 2 °C). The loading rate was 1 mm·min^−1^. The compression experiment was carried out up to the strain values of about 50% that was found for the compositions to be still in the region, where the loaded samples will completely recover their shape and volume after loading. On the contrary, at least for the representative sample polySMA-0.5, the values of strains above 53% were found to be on the verge of permanent mechanical failure. Form obtained stress-strain curves, the values of Young’s moduli in uniaxial compression mode (*K*) and the compressive stresses (*σ*) corresponding to several strain values (10%, 30%, and 40%) were determined. For each sample composition, ten specimens were measured. The values of recorded properties are given as averages from eight specimens ± standard deviations (maximal and minimal values of the set of ten values were not counted).

#### 2.3.4. Thermal Properties

The thermal properties of polySMA xerogels were studied by differential scanning calorimetry (DSC) using a DSC Q2000 (TA Instruments, Inc., New Castle, DE, USA). The measurements were performed in the temperature range from −70 to 200 °C at a heating rate of 10 °C·min^−1^ in nitrogen (flow rate 50 cm^3^·min^−1^). The glass transition temperatures (*T*_g_) were determined from the second heating scans.

The thermal stability was examined by thermogravimetric analysis (TGA) using a thermogravimeter Pyris 1 TGA (Perkin Elmer, Inc., Waltham, MA, USA) in the temperature range from 30 to 650 °C at a heating rate 10 °C·min^−1^ under a nitrogen flow 25 cm^3^·min^−1^.

#### 2.3.5. Determination of the Low Molecular Residue in Hydrogels

Polymer samples of approximately 1.5 g were immersed into 20 mL of PBS solution (pH 7.4) or into 20 mL of ethanol and shaken at 37 ± 1 °C in a laboratory shaker (Incubating Shaker IKA KS 4000i, 135 rpm, Fisher Scientific, Prague, Czech Republic). After 40 days, aliquots of the solutions were analyzed to determine the content of the extracted low molecular residues after polymerization, i.e., monomers, crosslinker and initiator. The samples obtained directly after polymerization (without acetone washing treatment) and samples washed after polymerization in acetone were examined in the extraction experiments.

The quantification of the extracted unreacted monomers was conducted by a HPLC-UV method using a Shimadzu LC-20AD (Shimadzu technology, Kyoto, Japan). The analysis was performed on a MERCK Chromolith RP-18e column (Merck, Prague, Czech republic) under the following conditions: Mobile phase ACN/water (75/25 *v*/*v*), flow rate 2.5 mL·min^−1^, and injection volume 10 µL. The wavelength of UV detection was set at 245 and 205 nm for styrene and MA, respectively. All experiments were made in triplicates and relative standard deviation was below 5%.

### 2.4. In Vitro Biocompatibility Test

Biocompatibility of the polySMA-0.7 hydrogel was evaluated using a human fibroblast cell line. The samples of approx. size of 8 mm × 4 mm × 2 mm were sterilized using a stream sterilizer at 120 °C for 30 min and put into 24-well plates with 1 mL culture media CellGRO containing ca 6 × 10^4^ human fibroblasts. The cells were cultivated with samples for 12, 24 and 36 h. The number of viable cells was determined by AlamarBlue Assay. The samples after particular time were withdrawn and AlamarBlue reagent was added to the medium with cells and incubated for 4 h at 37 °C. The viable/metabolically active cells reduce the active component of the AlamarBlue reagent resazurin to resorufin, whose fluorescence was detected in a Synergy Neo plate reader (BioTek, Moravska Ostrava, Czech Republic) using an excitation at 570 nm and an emission at 600 nm. The fluorescence intensity directly correlates with the number of viable cells. As a control, the cells cultivated in medium without samples were employed. Each time point was conducted in triplicate, the average values and standard deviations were calculated.

### 2.5. In Vivo Experiments

Animal studies were approved by the Committee for Animal Care and Use of The First Faculty of Medicine in Prague. In order to study the tissue reaction, the hydrogel expanders were implanted intramuscularly into the dorsal muscles of BALB/c mice (Velaz, Prague, Czech Republic). The experimental animals were anesthetized with isoflurane and the skin, subcutaneous tissue and muscle was cut. The implants (of approx. size of 8 mm × 4 mm × 2 mm) were carefully inserted into the dorsal muscle and the wound was sutured layer by layer. The animals were given analgesics and antibiotics after surgery. 3, 5, 7, 18 and 31 days after implantation the mice were sacrificed (3 animals in each group, each animal having implant in right and left leg), the implant was removed, measured and weighed. Surrounding tissue was fixated with paraformaldehyde and stained for Hematoxylin & Eosin (Sigma-Aldrich, Prague, Czech Republic). The thickness of connective tissue capsule around the implant was measured by the image analysis software (Zeiss, Prague, Czech Republic).

### 2.6. Statistical Analysis

Statistical analysis was performed in Origin 2019 64 Bit using one way ANOVA test. A value of *P* < 0.05 was considered significant.

## 3. Results and Discussion

In the present work, crosslinked copolymers of styrene and MA were synthesized and examined in respect to their potential application as soft hydrogel TE. The strictly alternating character of the styrene-MA copolymers as usually obtained under the conditions of their radical copolymerization [25] results in polymer chains of high structural regularity [26] and an interesting amphipathic nature: The polySMA can be dissolved in polar such as alcohols, even aqueous as well as in nonpolar or weakly polar organic solvents such as tetrahydrofuran or ethyl acetate. The behavior of radically polymerizing MA monomer and its reactivity towards the styrene comonomer is driven by the electron resonance stability on its double bond. The MA is known not to homopolymerize but it copolymerizes with the other monomers of the opposite electron polarization, e.g., vinyl monomers such as styrene.

In aqueous medium at presence of ions, MA units incorporated in the polySMA chains undergo hydrolysis leading to the formation of carboxylate groups (Figure 1b). As a consequence, the hydrophilic character of the polySMA-based macromolecular network increases with the degree of MA hydrolysis, which results in a further absorption of water inevitably accompanied with an increase of the hydrogel volume in later stage of swelling process.

In this study, the equimolar ratio of these two monomers was used in all prepared hydrogels. The DVB was selected as a crosslinking agent as it is a structural analogue of styrene and it copolymerizes readily with the both monomers. In order to find optimal concentration of the crosslinker, we made preliminary polymerization and swelling experiments. The hydrogels prepared with less than 0.5 mol % of the DVB showed very fast growth of volume upon swelling in PBS, whereas the hydrogels containing more than 1 mol % of DVB were in their swollen state weak and mechanically failing even in the initial stage of swelling. Therefore, in this study, the DVB crosslinker content was set in the range between 0.5 and 1.0 mol %.

### 3.1. Swelling Measurements

The swelling phenomenon of macromolecular covalent network entails macroscopic change of sample volume: Mere swelling that is not complicated by simultaneous extraction is represented as volume growth to certain maximum equilibrium value. The equilibrium volume fraction of the given network-solvent system is a unique physico-chemical parameter that is related to the macromolecular network structure, namely its crosslink density, and to chemical composition of the system.

**Figure 2:**
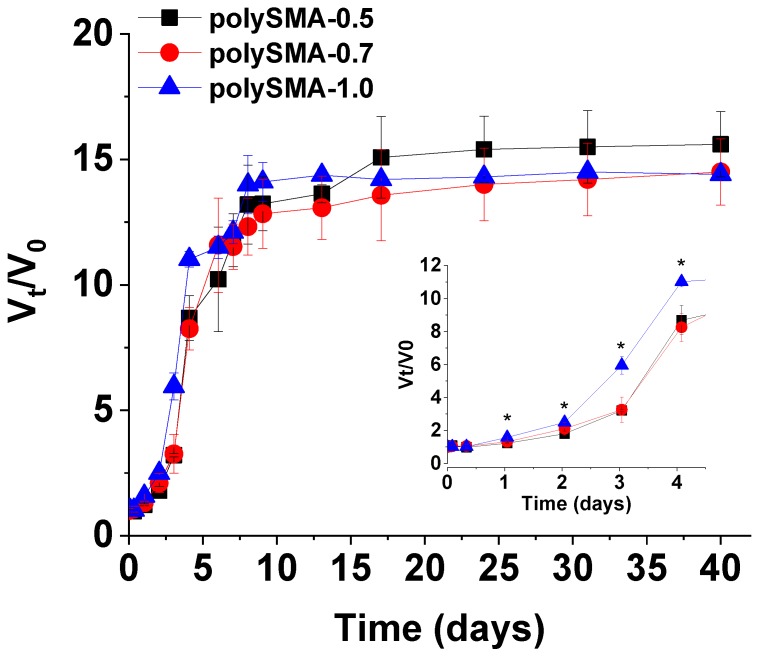
The time-dependence of swelling volume ratio (*V*_t_/*V*_0_) in PBS solution (pH 7.4) of the polySMA hydrogels prepared with different crosslinker content. Error bars were calculated using standard deviation (*n* = 3). The values with * represent the significant difference between the swelling ratios of individual hydrogels with *P* < 0.05.

The time-dependent swelling profiles of the polySMA hydrogels in PBS solution that represent aqueous environment containing ions (PBS osmolality is approx. 300 mOsm/kg aq) are shown in Figure 2 as the changes of the volume degree of swelling *V*_t_/*V*_0_. In these gels, the physical swelling is accompanied by a chemical change of the macromolecular network: The MA groups undergo hydrolysis and the anhydride is converted into carboxyl groups. Although the volume growth of the polySMA samples seems to be quite fast, there is an initial “induction” period of approx. two days when the volume change is small for all tested samples (see insert in Figure 2). After this induction period that is related to both, the hydrolysis and also to the process of transition of the macromolecular network from its glassy to rubbery state, the swelling course becomes somewhat auto-accelerated–the linear portion of swelling volume change between the days 2 and 8. The fast solvent uptake is caused by more intensive hydrolysis enabled by the opening of the network structure and higher availability of hydrolyzing MA units at a time. The enhanced swelling rate is also related to the increasing segmental mobility of the network due to the drop of the mean temperature of glass transition (*T*_g_) of the system polySMA-swelling liquid. The already hydrolyzed MA units further hydrophilize the polymer and also make the polymer chains anionic due to deprotonation of the formed carboxylates, which probably both contributes to the network expansion within this period by increased chain hydration and Coulombic repulsion among the polymer chains. Last but not least, as acid is formed during anhydride hydrolysis, mildly acidic environment inside the hydrolyzing gel probably autocatalytically accelerates hydrolysis of the remaining anhydride groups. This process is facilitated by the higher mobility of the network chains in the rubbery state. The volume degree of swelling reached its maximum after approx. 30 days for all the hydrogel compositions attaining the values more than 14, with the highest value being 15.7 for the polySMA-0.5 sample.

The maximum swelling of the polySMA hydrogels is surprisingly high; the ratio of 15.7 corresponds to the content of gel only 6.4 vol % for the polySMA-0.5 composition. The other two gels swell somewhat less, their gel content being around 7 vol %, still remarkably low. The crosslinker content was found to significantly influence the swelling course (*P* < 0.05) within 1 to 4 days. At the initial stage (up to day 1) and after day 4, the differences between swelling ratios were not statistically significant (Figure 2). As we already pointed out, when ionized, the anhydride forms carboxylate groups and thus hydrophilicity of the whole structure increases. At a given ionic strength and pH, certain equilibrium degree of ionization is attained (see Figure 1b). All MA units in polySMA chains are ionized at pH as high as 12–14 [27]. In our case, the polySMA gels swell in the PBS solution of pH 7.4 and containing several anions and cations that participate in the hydrolysis. The detailed mechanism of the hydrolysis of MA units in these networks is still not fully understood and is under research interest [28].

From the practical application point of view, the approx. 2 days lasting induction period without rapid swelling would be particularly useful in the surgical application of these materials as tissue expanders. If the material expands immediately after placed in contact with tissue fluids, it brings a high risk of tissue rupture at the incision place. In case of postponed swelling, the wound has the opportunity to partly heal and the risk of overpressure damage by the swelling gel is noticeably lower.

**Figure 3:**
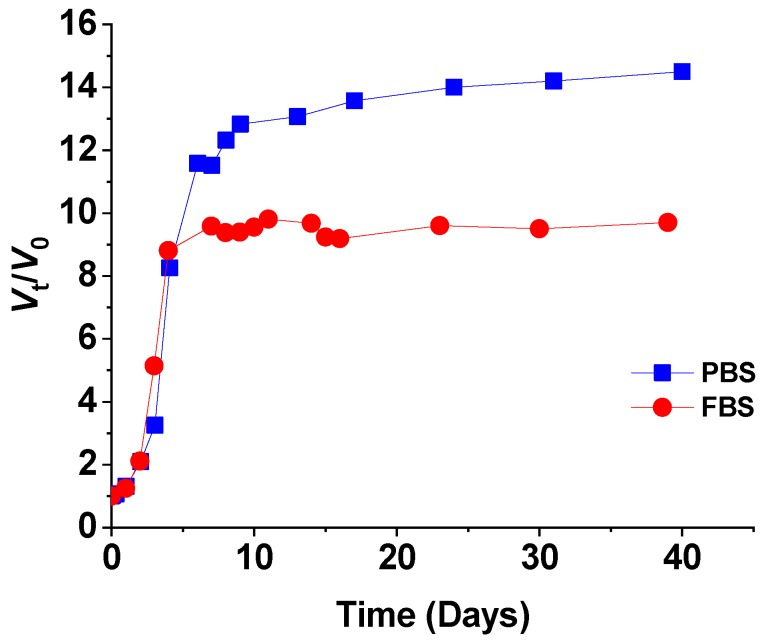
The time-dependence of swelling volume ratio (*V*_t_/*V*_0_) of the hydrogel polySMA-0.7 in PBS and FBS.

To better estimate the swelling process in living tissue, swelling behavior of polySMA-0.7 sample was measured in FBS and compared to swelling in PBS solution as shown in Figure 3. It is evident that until the day 5 the swelling profiles are comparable. With increasing time, the sample increased its volume more in PBS reaching the *V*_t_/*V*_0_ value 14.5 compared to 9.7 in FBS having the osmolality as the PBS solution. The difference in swelling is probably caused by additional covalent crosslinking of the polymer chains by proteins from FBS (which typically contains several primary amino groups on the lysine residues per protein macromolecule able to react with MA units). The smaller volume increase in FBS is, however, still sufficient for the intended application and it can be stated that swelling profiles measured in PBS solution provide relevant information for the estimation of swelling in in vivo conditions.

**Figure 4:**
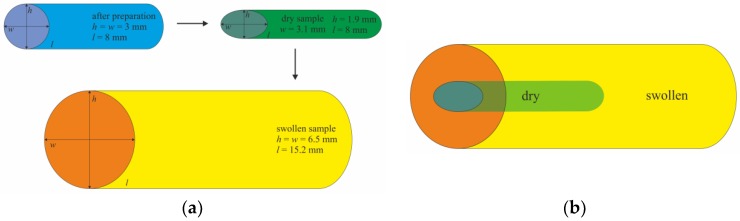
Illustrative images: (**a**) hydrogel anisotropic drying-swelling behavior; (**b**) comparison of the sizes of dried and swollen samples.

Another interesting feature of the prepared polySMA hydrogels is their anisotropic drying–swelling behavior, a kind of shape memory, that can be achieved by drying the swollen samples, obtained after polymerization in excess of ethyl acetate, that leads to fixing the sample shape by vitrification. Swelling of such dried samples leads to anisotropic increase in a particular dimension. In this study, the samples were prepared as cylinders with a circular cross-section (determined by the inner diameter and shape of the polyethylene tubes used as molds for polymerization). After washing and drying the samples after polymerization, the samples gained flattened cross-sections. Upon swelling, the former circular shape of the sample cross-section restored (Figure 4). The relative changes of the samples in particular dimensions, i.e., height, width and length, expressed as ratio a_sw_/a_0_ are given in Table 1**,** where a_sw_ represents the dimension after 40 days of swelling and a_0_ the dimension of the dry, extracted sample before swelling. The obtained values are the averages ± standard deviations (*n* = 3). Height of the samples increased more than three times, while width and length increased less than twice. The possibility of setting up a specific volume change in selected direction is quite desired in restoration surgery.

### 3.2. Crosslink Density and Mechanical Properties

The theoretical crosslink density *ν*_id_ of prepared polySMA hydrogels was calculated from the composition of the polymerization mixture (Equation (2)) and the values are given in Table 2. Mechanical properties were characterized in compression mode and the data served for estimating the values of apparent crosslink density *ν*_e_. The values of *ν*_e_ together with values of Young’s moduli and compressive stresses are also given in Table 2.

There were no significant differences between the values of Young’s moduli and compressive stresses depending on the crosslinker content. This is due to the experimental scatter, the relatively narrow range of crosslinker concentrations and the fact, that the gels were not fully swollen to their maximum volume because of not complete ionization of MA units. The nonionized portions of the polySMA structure function as the additional physical crosslinks constraining further volume expansion.

The values of the compressive stresses at both low and medium strains are quite high with respect to significantly high value of swelling. Also, these gels could withstand quite high values of compressive strains: they bore stresses up to 50% without any mechanical failure.

To give a comparative example, we synthesized a hydrogel based on poly(vinylpyrrolidone) containing 0.5 mol % of crosslinker 3’-ethylidene-bis(*N*-vinyl-2-pyrrolidone). This hydrogel swells in water to *φ*_2_ ≅ 40, which is much higher network structure content compared to polySMA hydrogels, but this system provides much lower values of the mechanical parameters: its modulus of elasticity is about 135 kPa, and the stress at strain 15% is 24 kPa. This gel breaks under the load of 20 kPa at strain of 30%. The higher mechanical strength of the polySMA hydrogels is explained by contribution of additional physical interactions between the styrene and MA units.

### 3.3. Thermal Properties

The thermal resistance of the prepared materials is important for the polymerization process. Polymerizations with a thermal initiation are carried out at elevated temperatures. Since it is an exothermic reaction, local overheating may occur and the material may be heated above the polymerization temperature (set up at 65 °C). Results from DSC and TGA measurements are given in Table 3. The TGA results indicated that any mass loss did not occur before 150 °C, thus far above the polymerization temperature (65 °C). Initial temperature of the main degradation step was in the range of 325–327 °C, which was significantly higher than the temperatures at which the materials were exposed during polymerization and drying.

The glass transition temperature, *T*_g_, is an important parameter for material processing. Due to the intended application, it is necessary to sterilize the materials (minimal sterilization temperature is 120 °C). If *T*_g_ is lower or similar, undesirable shape changes may occur during sterilization process. The prepared copolymers polySMA exhibited *T*_g_ values between 145 to 145 °C, safely above the sterilization temperature. In general, the thermal resistance of the polySMA copolymers crosslinked by DVB in the range of 0.5–1.0 mol % is sufficient for the preparation and processing of these materials for the intended application.

### 3.4. Residua in the Materials after Polymerization

Amount of residual monomers in prepared hydrogels was quantified for copolymer polySMA-0.7. Samples were immersed in PBS or ethanol for 40 days and the concentration of unreacted styrene and MA in the solutions was quantified by HPLC-UV with the limit of detection approx. 1 × 10^−6^. The contents of residua in samples after the polymerization (without any treatment) and in samples washed in acetone are given in Table 4. The released styrene residua were detected only in samples without treatment in acetone soaked into the ethanol and their amount was quite low, confirming of almost complete consumption of styrene units. Unreacted styrene did not release into PBS even in case of previously unwashed samples. MA released from all samples to both ethanol and PBS in significant amount. Almost 17% of initial amount of MA was released into PBS and more than 6% to ethanol from the samples that were not treated in acetone. Treatment in acetone decreased these values to 0.08% measured in water and 0.06% in ethanol.

Radicals of both monomers have high electron resonance stability, in combination with the opposite polarization, they tend to alternate, which is expressed by copolymerization parameters close to zero. Copolymerization parameters of styrene and MA are 0.41 and 0.01, respectively [29]. The tendency to the alternate copolymerization is increased by the steric hindrance of the MA molecule, whose homopolymerization is therefore very limited. However, the results acquired from measuring of residual low molecular compounds in the materials imply that the polymerization of styrene with MA was not strictly alternating and incorporation of the styrene units into the polymer was faster in comparison with MA, which then remain unreacted in the polymer.

This careful analysis of the unreacted residues revealed that under the selected polymerization conditions, at presence of ethyl acetate diluent, a certain amount of styrene units must had homopolymerize most likely as styrene blocks within the otherwise alternating network. So, this unusual course of styrene and MA polymerization is likely due to the ethyl acetate environment in which the polymerizing system is diluted to 35 vol %. The further investigation of this “classical alternating” monomer pair tendency to “styrene homoblocking” behavior is underway.

The tendency of styrene segments to form associate (either in solution or in melt) has been studied and utilized for increasing toughness of polymers [30]. This can be the reason for the enhanced compressive resistance of the swollen polySMA hydrogels as was described above in the Section 3.2.

The hydrogels prepared by free-radical polymerization with subsequent washing in acetone did not released any detectable residua of styrene, the values of MA residues determined by HPLC into the water and ethanol do not pose a risk of cytotoxicity in in vivo application as a tissue expanders, which was confirmed by the experiments on cell lines and animal model discussed below.

### 3.5. Biocompatibility Test

**Figure 5:**
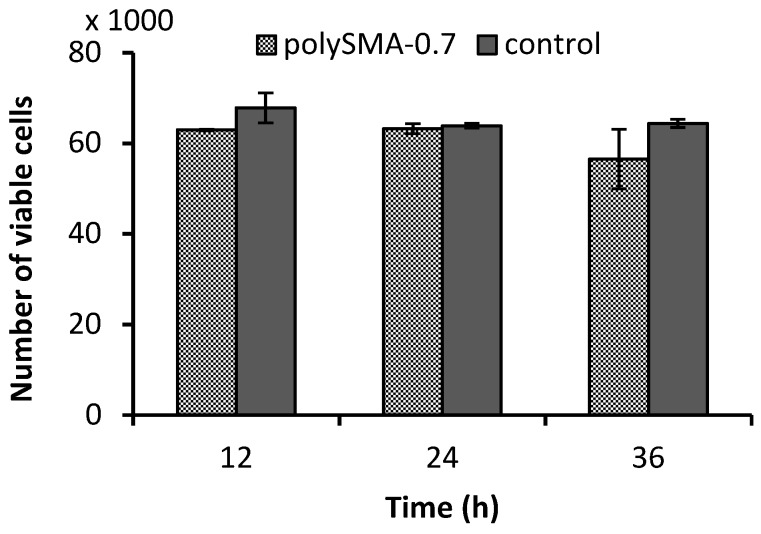
Number of viable human fibroblast cells after various time of their growth on polySMA-0.7 hydrogel determined by AlamarBlue assay. Error bars were calculated using the standard deviation (*n* = 3).

Determination of the sample cytotoxicity in the presence of cell line provides information about toxicity of the material for intended in vivo application. The biocompatibility test was performed using human fibroblasts. A samples weighing approx. 0.1 g were immersed in 1 mL of media with cells. The number of growing/viable cells after 12, 24, and 36 h incubation time with polySMA-0.7 sample was monitored. The time dependent influence of the sample presence on cell growing compared to the control test (cells without sample) is shown in Figure 5. It is apparent that the differences in number of viable cells between polySMA-0.7 hydrogel and control are not significant providing noncytotoxicity of tested polymers.

### 3.6. In Vivo Test

**Figure 6:**
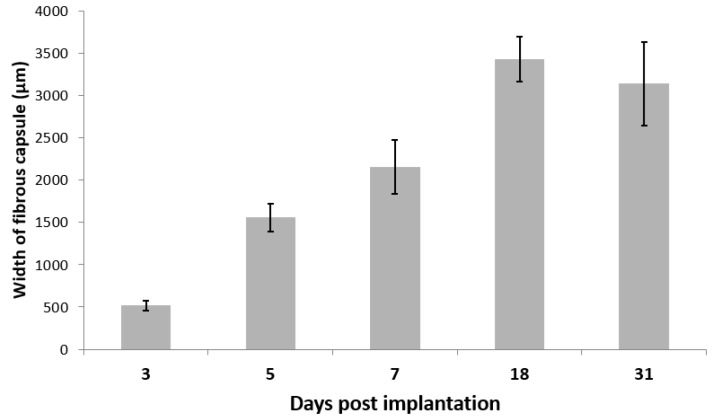
The size of the connective tissue capsule around the implant Error bars were calculated using the standard deviation (*n* = 6).

**Figure 7:**
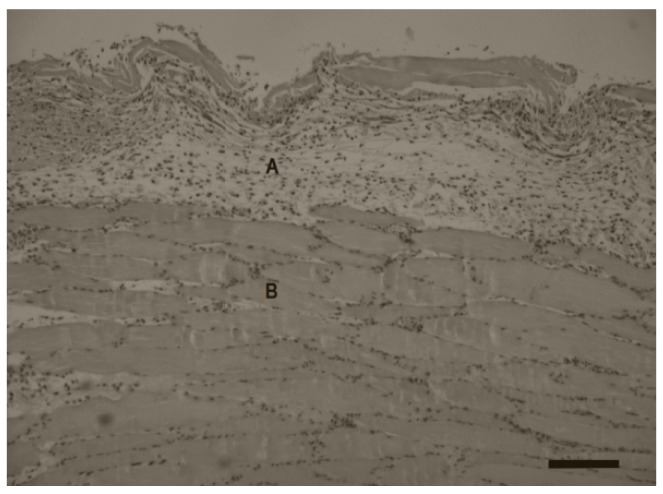
Hematoxylin & Eosin stained tissue section of the connective tissue capsule around expander implanted for 7 days. (**A**) Connective tissue capsule formed around implant, (**B**) muscle tissue. Scale bar = 0.5 mm.

The histopathologic observation after implantation of polySMA-0.7 hydrogels in mice was performed and evaluated by macrography and microscopy. All of the implants were surrounded with fibrous capsule formed by the growth of the implant; with the time of sample implantation the size of capsule increased (Figure 6). We observed no signs of necrosis in the border between the implant and the connective tissue capsule. There was visible small cellular infiltration around the implants (acute inflammatory response), however we observed no macrophages, foreign body giant cells nor granuloma formation around the implants (Figure 7). The sizes of the implants were measured immediately after their removal and the volume expansion expressed as volume ratio *V*_t_/*V*_0_ is given in Table 5. The volume ratio increased from 8.7 on day 3 to 12.7 on day 31 showing thus higher initial volume expansion compared with the values determined in vitro in PBS or FBS (see Figure 2 and Figure 3) at corresponding swelling time.

It can be expected some difference between the in vitro and in vivo swelling caused by different environments and spatial constraints in the body. In this study, the in vivo experiments provided rather incipient guidance and valuable evidence that the polySMA expanders are biocompatible and that they do not induce necrosis. The swelling course of the hydrogel can be further tuned up (slown down) by the choice of polymer composition (partial substitution of hydrolyzing comonomer MA by structurally similar but nonhydrolyzing one), or by the crosslinker type and content (more flexible crosslinker at a higher concentration). Perhaps quite an interesting approach to solve this issue is to use the expanders pre-swollen in vitro in a physiological solution to a desired swelling degree before inserting them in vivo. This simple pre-swelling treatment can help to optimize the gel volume growth window for the healing process.

## 4. Conclusions

We have shown that the covalently crosslinked polySMA alternating copolymer hydrogels are well-suited materials for TEs mainly due to their protracted swelling and excellent biocompatibility. The favorable swelling profile with protracted volume growth resulted from the ongoing hydrolysis of MA units in the polymer network chains during swelling. The swelling course and equilibrium can be further adjusted by *p*-divinylbenzene crosslinker content and thus the gels can be made to meet the requirements of given application. Hydrogels provide sufficient final swollen volume with around 15 times of their initial volume under the free swelling conditions and 12.7 times in constrained in vivo swelling conditions while still maintaining their structure integrity. In the free swelling in PBS solution, the gels exhibited a two-day initial induction period caused by transition from glassy state paralleled with gradual hydrolysis of MA units accompanied with only a small volume change. This feature is favorable for surgical applications because the initial delay in volume growth allows the wound to heal and thus diminishes the risk of rupture at the sutured site. The mechanical measurements showed that the polySMA hydrogels exhibited relatively high compressive stresses (380 to 550 kPa at 40% strain) and withstood compressive strains up to 50% without any mechanical failure, although their swelling was high (*φ*_2_ ≅ 0.065). In vitro tests with human fibroblasts demonstrated the biocompatibility of prepared hydrogels and, moreover, in vivo tests showed no tissue inflammation or necrosis, confirming that no toxic components released into the body after implantation into mice. Taken together, the results constitute the polySMA copolymers as promising soft hydrogel TEs for tissue restoring applications, not only in stomatology. However, swelling expansion determined after in vivo implantation seems to be rather fast so the future challenge remains the design of materials that exhibit a slower, close to linear volume expansion over the first 4 to 5 days. These aspects will be a topic for future research.

## Figures and Tables

**Table 1:** Dimension ratios of hydrogel polySMA-0.7 after swelling in PBS.

Dimension	a_sw_/a_0_^1^
height	3.29 ± 0.04
width	1.92 ± 0.03
length	1.84 ± 0.03

^1^ Average value ± standard deviation (*n* = 3).

**Table 2:** The polySMA hydrogel characteristics: Equilibrium volume ratios in PBS solution, crosslink density and mechanical properties.

Sample	Swelling in PBS Solution at RT		Crosslink Density		Mechanical Properties–Compression Test	
	*V*_t_/*V*_0_	*φ*_2_^1^	*ν*_id_ (mol cm^−3^) ^2^	*ν*_e_ (mol cm^−3^) ^3^	*K* (kPa)	*σ* (kPa) ^4^
polySMA-0.5	15.7 ± 1.3	0.064	1.10 × 10^−4^	2.50 × 10^−4^	746 ± 58	451 ± 64
polySMA-0.7	14.5 ± 1.3	0.069	1.54 × 10^−4^	2.98 × 10^−4^	855 ± 44	556 ± 31
polySMA-1.0	14.4 ± 0.2	0.069	2.20 × 10^−4^	2.78 ×10^−4^	796 ± 88	379 ± 41

^1^ Equilibrium volume fraction of gel from volumetric swelling experiment expressed according to Equation (1). ^2^ Crosslink density calculated from composition according to Equation (2). ^3^ Crosslink density calculated from compression modulus according to Equation (3). ^4^ Compressive stress at 40% strain.

**Table 3:** Thermal properties of polySMA hydrogels.

Sample	*T*_g_	*T*_onset_	DTGA_max_	W_R600 °C_
	(°C)			(wt %)
polySMA-0.5	145	325	398	10
polySMA-0.7	149	327	401	10
polySMA-1.0	146	326	404	11

*T*_g_—glass transition temperature. *T*_onset_—the initial temperature of the main degradation step. DTGA_max_-temperature at which degradation is the fastest. W_R600 °C_—content of noncombustible residuum in a nitrogen atmosphere at 600 °C.

**Table 4:** The amounts of residual monomers leached into PBS and ethanol from sample polySMA-0.7 determined by HPLC.

Leaching Medium	Sample Status	Amounts of Leached Residual Monomer (wt %)	
		Styrene	MA
PBS	Unwashed	0.00	16.94
	Washed in acetone	0.00	0.08
Ethanol	Unwashed	1.89	6.22
	Washed in acetone	0.00	0.06

**Table 5:** The volume ratio (*V*_t_/*V*_0_) of polySMA-0.7 expanders removed after certain time of the implantation into rat legs.

Time (days)	*V*_t_/*V*_0_^1^	
	Right Leg	Left Leg
3	8.8 ± 0.3	8.7 ± 0.2
5	11.1 ± 1.1	11.2 ± 0.3
7	11.0 ± 2.2	10.7 ± 0.2
18	12.5 ± 0.4	11.6 ± 1.1
31	12.6 ± 1.4	12.7 ± 1.1

^1^ Average value ± standard deviation (*n* = 3).
